# Increasing resilience to stress by home-based transcranial stimulation

**DOI:** 10.21203/rs.3.rs-7865004/v1

**Published:** 2025-10-17

**Authors:** Rubén Romero-Marín, Davide Cappon, Simon Fankhauser, Javier Solana-Sánchez, Ruben Perellón-Alfonso, Josep Maria Tormos-Muñoz, Luiz Pessoa, David Bartrés-Faz, Gabriele Cattaneo

**Affiliations:** Institut Guttmann, Institut Universitari de Neurorehabilitació adscrit a la UAB, Badalona, Spain; Department of Neurology, Harvard Medical School, Boston, MA, USA; Institut Guttmann, Institut Universitari de Neurorehabilitació adscrit a la UAB, Badalona, Spain; Institut Guttmann, Institut Universitari de Neurorehabilitació adscrit a la UAB, Badalona, Spain; Institut Guttmann, Institut Universitari de Neurorehabilitació adscrit a la UAB, Badalona, Spain Departament de Medicina, Facultat de Medicina i Ciències de la Salut, i Institut de Neurociències, Universitat de Barcelona, Barcelona, Spain Institut d’Investigacions Biomèdiques August Pi I Sunyer (IDIBAPS), Barcelona, Spain; Centro de Investigación Traslacional San Alberto Magno, Universidad Católica de Valencia San Vicente Mártir, Valencia, Spain; Department of Psychology, University of Maryland, College Park, MD, USA; Departament de Medicina, Facultat de Medicina i Ciències de la Salut, i Institut de Neurociències, Universitat de Barcelona, Barcelona, Spain; Institut Guttmann, Institut Universitari de Neurorehabilitació adscrit a la UAB, Badalona, Spain

**Keywords:** tDCS, HB-tDCS, stress, resilience

## Abstract

Mental health disorders, especially anxiety and depression, affect nearly one billion people worldwide, with chronic stress playing a major role in their onset and severity. Despite growing demand, access to care remains limited, underscoring the need for scalable, preventive interventions. This study investigates the feasibility, safety, and preliminary effects of a Home-Based transcranial Direct Current Stimulation (HB-tDCS) protocol targeting the left dorsolateral prefrontal cortex (L-DLPFC) to enhance stress resilience in healthy middle-aged adults. Thirty-one participants completed ten sessions of self-administered tDCS over two weeks, monitored remotely. Adherence was high (98%), with minimal technical issues and no serious adverse events reported. Mild, transient side effects occurred in only four participants. A stress induction paradigm involving pupillometry, electrodermal activity (EDA), and subjective stress and anxiety ratings was administered pre- and post-intervention. Results indicated a significant reduction in acute stress appraisal, but no significant changes in anxiety scores or physiological markers. These findings support the feasibility and safety of HB-tDCS and suggest potential stress-reducing effects, though the absence of a control group and limited psychophysiological impact warrant cautious interpretation. Future research should explore longer stimulation protocols, controlled trials, and clinical applications in populations with heightened stress vulnerability.

## Introduction

The World Health Organization (WHO) reports that mental disorders, primarily anxiety and depression, affect approximately 1 billion people globally. This isn’t just a statistic; it’s a call to action (Vos et al., 2020). Mental health challenges disrupt relationships, hinder academic and professional pursuits, diminish overall well-being, and drastically increase mortality risks (Berghöfer et al., 2020; Fagiolini & Goracci, 2009; Saarni et al., 2007; Walker et al., 2015). Despite the alarming prevalence of mental health disorders, up to 70% of the population remains untreated due to factors such as inadequate resources and pervasive social stigma surrounding mental health (Henderson et al., 2013). Demand for mental health services has surged, exceeding available care, especially in the aftermath of COVID-19 (Zhu et al., 2022) stressing the need for urgent global action to address mental health (GBD 2019 Mental Disorders Collaborators, 2022).

Within the landscape of mental health, stress is regarded as one of the primary risk factors, closely related to the development of various psychiatric disorders. Defined as the body’s response to demanding or threatening situations (Selye, 1936), stress transcends mere physiological arousal, exerting profound effects on mental well-being (Slimmen et al., 2022). Chronic exposure to stressors heighten vulnerability to anxiety (Daviu et al., 2019), depression, and post-traumatic stress disorder (PTSD), both precipitating their onset and exacerbating their severity (Davis et al., 2017). Repeated and chronic exposure to stressors can cause hippocampal atrophy (Belleau et al., 2019; Bremner, 1999, 2006; Mcewen, 2004; McEwen, 2005; McEwen et al., 2016), prefrontal cortical dysfunction (Belleau et al., 2019; Jimoh & Omiyefa, 2025; McEwen, 2005; McEwen et al., 2016), and amygdala overactivation (Bremner, 2006; McEwen et al., 2016; Piccolo et al., 2018), leading to memory impairments (Bremner, 1999; Kim & Kim, 2023), executive function and impulse control problems (Bremner, 2006; McEwen, 2005), and affective and emotional instability (Jimoh & Omiyefa, 2025; Mcewen, 2004); it increases cortisol levels (Kim & Kim, 2023), which impair neurogenesis and cause neuronal damage (Caruso et al., 2018); contributes to hypertension and raises the risk of cardiovascular disease (Ezkurdia et al., 2023; Xu & Shi, 2025); alters immune function, increasing the risk of infections (Berlanga-Acosta et al., 2020; Maciejczyk et al., 2019; Patel & Edison, 2024); leads to weight gain and contributes to insulin resistance, increasing the risk of Type 2 diabetes (Verdile et al., 2015; Xu & Shi, 2025); etc. In patients with Alzheimer’s disease (AD) and other neurodegenerative disorders, uncontrollable stress accelerates dementia and disability (Barone et al., 2021; Caruso et al., 2018; Ezkurdia et al., 2023; Terzo et al., 2021).

WHO advocates for preventive interventions promoting resilience to stressors to prevent or delay the onset of mental health-related disabilities (Pascual-Leone & Bartres-Faz, 2021). Resilience to stressors is a well-established concept, highlighting that not all individuals develop the same mental or behavioral health consequences when exposed to the same stressor (Perellón-Alfonso et al., 2022). Resilience refers to the processes that empower individuals to withstand symptoms, disability, or distress development (Labrague, 2021).

Recent advancements in behavioral paradigms have paved the way for experimentally study the neural mechanisms of resilience to stressors (McMenamin et al., 2014; Limbachia et al., 2021) by monitoring brain neurophysiological responses via functional MRI or EEG, measuring the electrical conductance of the skin (Limbachia et al., 2021; Meyer et al., 2019), heart rate variability (HRV; Maheshkumar et al., 2016; Szakonyi et al., 2021), or pupillometry (Schneider et al., 2020; Suárez-Pereira et al., 2022; Winn et al., 2018). In parallel, transcranial direct current stimulation (tDCS) has emerged as a promising method for non-invasive modulation of neural circuits implicated in resilience to induced stressor (Castelo-Branco & Fregni, 2020). By applying low-amplitude electrical currents to specific brain regions, tDCS holds potential for rebalancing aberrant circuitry and ameliorating symptoms associated with mental illness and stress (Su et al., 2024; Yamada & Sumiyoshi, 2021; Antal et al., 2017). Promising initial results suggest potential benefits of tDCS in reducing physiological surrogates of stress, especially heart rate variability (HRV) and salivary cortisol levels (Brunoni et al., 2013; Montenegro et al., 2011). Direct effects on stress symptoms have also been reported, showing the potential for the prevention of stress-induced mental disorders (Bogdanov & Schwabe, 2016).

The prefrontal regions of the brain have consistently emerged as pivotal targets in tDCS interventions designed to alleviate psychosocial stress and common psychiatric disorders. Carnevali et al., (2020) present a compelling evidence-based hypothesis of a dual modulatory mechanism of tDCS, which encompasses cognitive control of stress and the autonomic system, involving predominantly parasympathetic (vagal) responses. The left dorsolateral prefrontal cortex (L-DLPFC) assumes a central role within the frontoparietal control network (FPCN), governing cortical and subcortical regions involved in emotion regulation. This network, implicated also in resilience against experimentally induced stressors, offers promising avenues for mitigating the detrimental effects of perceived stress on anxiety and depressive symptoms (Cabello-Toscano et al., 2022). However, the response to anodal left DLPFC stimulation can differ depending on individual anxiety traits (Sarkar et al., 2014), and assessment of specific psychological traits at baseline could help determine which individuals would benefit more from the effects of tDCS.

The conventional model of tDCS administration presents practical hurdles, requiring in-person sessions that may be inconvenient or inaccessible for many individuals. Home-Based tDCS (HB-tDCS) protocols represent a paradigm shift in the delivery of neuromodulation, offering a potential solution to address these limitations (Castelo-Branco et al., 2020). By empowering patients to self-administer tDCS within their own homes, this approach not only enhances accessibility but also promotes intervention adherence and engagement (Cappon et al., 2022). Initial efforts to develop and establish common practices to safely and effectively administer tDCS in the home environment have been conducted by Cappon et al., (2023).

Our study aimed to investigate the safety and feasibility of a remotely supervised, self-administered tDCS intervention designed to reduce perceived stress among middle-aged healthy adults. By assessing adherence, user experience, and safety parameters, we provide valuable insights into the potential of HB-tDCS as a viable intervention to reduce perceived stress and enhance stress resilience.

## Materials and methods

### 2.1 Participants

31 healthy individuals participating in the Barcelona Brain Health Initiative cohort (Cattaneo et al., 2020) took part in this study. Exclusion criteria included a history of neurological, psychiatric, or cardiac disorders, as well as any contraindications to tDCS such as implanted metallic devices or a history of seizures. Demographic characteristics are presented in Table 1.

Prior to participation, all eligible individuals provided explicit written informed consent after receiving detailed information about the study procedures and possible risks. The protocol was approved by the Ethics and Clinical Research Committee of the Catalan Hospitals Union (Comitè d’Ètica d’Investigació amb medicaments (CEIm) de la Fundació Unió, CEI 23/35), and registered as a clinical trial at clinicaltrials.gov (NCT06051071).

### 2.2 Procedures

Our primary aim was to study feasibility, safety and adherence, to an HB- tDCS stimulation protocol, while a secondary aim was to assess behavioral changes in stress response before and after stimulation. Participants completed a pre- and post-stimulation assessment, and underwent detailed remote monitoring of the stimulation protocol which consisted on 10 sessions of HB-tDCS (one session per day Monday to Friday for two consecutive weeks).

#### Primary outcomes: Feasibility, Safety and Adherence

To assess feasibility, we recorded the number of sessions in which HB-tDCS was halted, for example due to elevated electrode impedance. Additionally, we documented any issues encountered during each session, including instances where additional measures such as adjusting the electrode cap or applying more conductive saline solution were necessary. Furthermore, we monitored instances where participants required assistance from study staff and recorded the frequency of participants needing remote technical support.

Adherence was evaluated by calculating the percentage of total HB-tDCS sessions completed out of the predetermined total of 10 sessions. We divided the number of completed sessions by 10 and then multiplied the result by 100 to obtain the percentage of adherence. “Completed sessions” refer to the total number of HB-tDCS sessions successfully completed by the participant. Conversely, “Halted sessions” denote the number of sessions that were omitted, interrupted or otherwise not completed according to the study protocol. Additionally, participants provided qualitative feedback about the HB-tDCS course during the follow-up assessments.

To evaluate the safety of the protocol, we recorded any reported side effects throughout the entire study period. This included documenting any adverse experiences reported by participants, such as headaches, neck pain, scalp pain, sleepiness dizziness, sensations under electrodes (tingling, scalp burn and scalp redness). Participants were explicitly asked about specific side effects (see Table 2). Participants were prompted to indicate the intensity of each side effects by categorizing their experience as mild, moderate, or severe (see Table 2).

#### Secondary outcomes: Behavioral changes in perceived stress and anxiety levels induced by acute stressor paradigm

Pre and post assessments were conducted to assess the impact of the Home-Based stimulation protocol on perceived stress. Participants were evaluated on an adapted version of the moving-circles paradigm (Limbachia et al., 2021; Meyer et al., 2019), where two circles move around the screen, sometimes moving closer and at times moving away from each other. When the circles touch, participants are delivered a mild electric stressor. Functional magnetic resonance imaging of subjects exposed to this paradigm has revealed that when participants think they can control the duration of the stressor by pressing a button, stressor-related responses across a threat-related brain network are decreased (Limbachia et al., 2021). In our adaptation, participants were seated in a chair and instructed to focus on a black screen displaying the two circles moving across it. When the circles collided, a distressing event was triggered, involving a brief electric discharge to the hand accompanied by an unpleasant noise and a white flash on the screen. The duration of this stressor event ranged from 0.2 to 2 seconds. Electric discharges were administered using a Digitimer Constant Current Stimulator DS7A (Digitimer Ltd) through two electrodes (input and output) placed on the right hand.

Psychophysiological stress responses were assessed during the task using pupillometry and EDA. EDA were recorded using Biopac MP150 hardware (Biopac Systems Inc), with sensors placed on the second and third fingers of the left hand. Pupillometry data were collected using a Tobii Pro Nano Eye Tracker (Tobbi AB). The sampling rate was 1000 Hz for EDA and 60 Hz for pupillometry.

The preprocessing pipeline was developed in MATLAB and included the following steps:

1. Marker Alignment: Stimulus markers were temporally aligned with the pupillometry data by locating the nearest timestamp in the pupil stream for each marker. This allowed for accurate annotation of stimulus onset times for further analysis and visual review.
2. Correlation and Cross-Eye Imputation: The Pearson correlation coefficient between the left (lPupil) and right (rPupil) eye signals were calculated using only complete (non-missing) data. Two basic linear regression models were then fitted: one to estimate the left eye signal from the right, and another to predict the right from the left. These models were subsequently used to fill in missing values when data from only one eye was available, when one eye’s value was missing but the other was present, the missing measurement was inferred using the relevant model.
3. Padding of Missing Segments: To reduce edge artifacts during filtering, regions with missing values were padded—adding three samples before and five after each gap. This ensured smoother transitions in and out of missing segments.
4. Low-Pass Filtering: The pupil data were filtered using a second-order Butterworth low-pass filter with a 5 Hz cutoff frequency. Filtering was applied exclusively to valid data points, and the resulting filtered values were reinserted into the original signal, keeping missing values in place.
5. Z-Score Normalization: Lastly, both the left and right filtered pupil signals were standardized using z-score normalization, yielding signals with a mean of 0 and a standard deviation of 1. This step supports comparability across participants and time windows in the analyses that follow.

The preprocessing pipeline for the EDA signal was implemented in MATLAB and included the following steps:

1. Resampling and Interpolation: EDA signal was resampled to match a uniform 250 Hz time grid to ensure alignment with other physiological signals. Both the phasic and tonic components were interpolated using spline interpolation, which minimizes artifacts and preserves the dynamics of the original signal.
2. Temporal Trimming and Alignment: EDA data were trimmed to match the duration of the experiment by extracting the segment between the first and last recorded event markers. This ensured temporal alignment with the other signals, facilitating downstream multimodal analysis.
3. Signal Synchronization: To investigate potential delays between the stimulus and the EDA response, cross-correlation analysis was conducted between the stimulus trace and the phasic EDA component. The optimal lag corresponding to the maximum correlation was identified and used for visual inspection of temporal alignment.
4. EDA Decomposition: The EDA signal was preprocessed using a model-based decomposition into tonic and phasic components. The tonic signal reflects the slowly varying baseline level of skin conductance, while the phasic signal captures rapid, event-related fluctuations. These components were analyzed separately in subsequent stages.
5. Statistical Modeling: Linear regression models were fitted to quantify the relationship between the stimulus position and both phasic and tonic EDA components. This analysis helped characterize the extent to which changes in stimulus dynamics elicited electrodermal responses.

The preprocessing pipeline for the stimulus distance signal between circles was implemented in MATLAB and comprised the following steps:

1. Resampling to Uniform Time Grid: The raw circle distance signal, originally sampled at a non-uniform rate, was interpolated to a uniform 250 Hz time grid using linear interpolation.
2. Extraction of Stimulus Trajectory: The distance between moving circles was extracted from the relevant dimension of the original time series. This signal served as the primary stimulus trace to which physiological responses were later aligned.
3. Temporal Alignment and Cropping: The resampled signal was trimmed to match the time range defined by event markers, ensuring that only the period corresponding to the experimental task was retained. This step also enabled consistent alignment with other outcome measures.
4. Identification of Salient Stimulus Events: Additional stimulus markers were generated by detecting local minima in the circle distance trace, corresponding to moments of near-contact between stimuli without electrical stimulation. Peaks below a predefined threshold were identified using peak detection methods to annotate these critical moments for further analysis.
5. Correlation and Regression Analyses: The signal was used as a predictor in linear regression models to evaluate its relationship with various physiological outcomes, including pupil size, electrodermal activity, and heart rate. These models provided quantitative estimates of the strength and direction of stimulus-response coupling.

The entire task lasted for 7 minutes, during which participants were instructed to maintain their gaze on the screen and minimize blinking to facilitate accurate pupillometry measurements.

This task was administered both before the commencement and upon completion of all scheduled HB-tDCS sessions. Perceived stress and anxiety levels were evaluated immediately after the task using the post task subscale of the Stress Appraisals of Acute Stress (SAAS) scale (Mendes et al., 2007), as well as the state subscale (STAI-S) of the State-Trait Anxiety Inventory (STAI). The SAAS scale has been developed to be used in laboratory stress paradigms and measure demand and resource appraisals. The post task subscale is composed by 10 items and participants responded using a 7-point likert scale ranging from 1 to 7. Higher scores represent higher stress appraisal. The STAI state subscale, designed for clinical use, comprises 20 items. Participants respond using a 4-point Likert scale ranging from 0 to 3. Higher scores represent higher state anxiety.

#### Home-Based tDCS Protocol

Participants were equipped with essential tools and instructions for self-administering tDCS sessions at home. This included a tDCS device (Sooma tDCS^™^ device, Oy, Helsinki, Finland), electrode sponges and a headcap featuring two designated holds corresponding to the positions of electrodes F3 and F4 in the 10–20 EEG system, along with detailed guidelines for electrode placement.

The electrode holes in the headcap were color-coded, aligning with the colors of the electrode leads from the tDCS device. This color coordination ensures that each electrode lead is correctly attached to its corresponding electrode, thus mitigating the risk of inadvertent mismatching between the electrodes and leads. The anode was in F3, the cathode in F4, and current was delivered at 2mA intensity for 30-minute.

Participants received in-person, verbal instructions and hands-on training in the laboratory to ensure correct adherence to the stimulation protocol. They were asked to rigorously follow the procedure at home and to immediately report any adverse events or difficulties encountered during the sessions. They were provided with a daily questionnaire via email to facilitate this reporting process. Additionally, in the event of any reported adverse event, participants were promptly contacted via telephone to aid and manage the situation effectively. Our study followed current HB-tDCS safety recommendations including providing specifically tailored one-to-one 30 minutes training and remote support, in addition to ongoing compliance supervision and side effects monitoring (Cappon et al., 2022, 2023; Charvet et al., 2020).

The stimulation protocol consists of 10 sessions, each lasting 30 minutes, to be conducted daily, including weekends. The device automatically performs an impedance check both before and during the administration of the tDCS current. If the electrode impedance exceeds 15kΩ, the stimulation is automatically suspended to ensure safety and efficacy. The current intensity was gradually increased over a period of 30 seconds, maintained at the stimulation intensity for 30 minutes, and subsequently decreased over another 30-second period. Moreover, participants had the autonomy to halt the session if they feel uncomfortable with the stimulation. They could do so by pressing the single button on the device. If they chose to continue, participants could then simply press the same button to resume the session. All participants received thorough instructions on how to operate the device and manage any potential issues related to impedance. Additionally, a demonstration was provided beforehand to illustrate the proper set-up of the cap and device components. The assembly of the entire tDCS device involved placing the electrodes in the cap, matching the colors of the cables (red and black) with the corresponding colors of the cap’s holes. Next, the cables were connected to the device, aligning the colors accordingly. Then, the cap had to be positioned on the head, ensuring that the central line aligned with the nose. Finally, the button was pressed to initiate the impedance check. If everything was in order, the same button was then pressed to start the stimulation.

Throughout the study period, participants were remotely monitored via telephone and email to ensure compliance and to address any questions or concerns. Follow-up assessments were conducted to evaluate the safety, feasibility, and adherence of the HB-tDCS protocol.

### Statistical analysis

To evaluate feasibility, safety and adherence we performed descriptive statistics on various parameters. This included analyzing the number of completed sessions, halted sessions, dropouts, issues during sessions, unallowed starts due to impedance, number of sessions where participants required technical support, and the total number of technical support requests. Additionally, we examined the occurrence and severity of side effects, including headache, neck pain, scalp pain, sensations under electrodes, sleepiness, scalp burn, scalp redness, and dizziness. We also calculated the percentage of sessions associated with each side effect severity level (mild, moderate, severe).

Wilcoxon signed ranked test was conducted to compare the mean scores between pre- and post-treatment assessments for both the STAI-S and SAAS. Statistical analyses and visualizations were performed using JASP (JASP Team, 2019).

To evaluate the impact of the intervention on physiological and model-derived outcome measures, a series of repeated measures ANOVAs were conducted. These analyses tested for significant changes over time (Pre vs. Post intervention) across multiple dependent variables related to pupil diameter and electrodermal activity (EDA).

Physiological Measures (Pupil and EDA Amplitudes): Pupil amplitude and EDA amplitude were analyzed as anticipatory autonomic responses, computed as the area under the curve (AUC) in the seconds leading up to each event. Repeated measures ANOVA was used to test for time-dependent differences in these raw amplitude measures.

Model Fit Indices (R^2^ and Beta Estimates): Linear models were employed to assess the relationship between the physiological signals and a stimulus proximity signal. For each participant and condition, the coefficient of determination (R^2^) was calculated to represent the proportion of variance in the physiological signal explained by the stimulus. Additionally, beta estimates (EST) were obtained to quantify the strength and direction of this association. These indices were also subjected to repeated measures ANOVA to assess pre-to-post intervention changes.

Model Accuracy (Mean Squared Error): Mean squared error (MSE) was used as an indicator of the accuracy of the linear models. For the EDA signal, MSE values were log-transformed to improve normality before statistical testing. Repeated measures ANOVA was used to assess changes in model accuracy across time points.

Statistical Metrics Reported: For each ANOVA, the following statistics were reported: sums of squares (SS), degrees of freedom (DF), mean squares (MS), F-values, uncorrected p-values (p-unc), generalized eta squared (η^2^g) as a measure of effect size, and the sphericity correction factor (ε), where applicable.

## Results

### 3.1 Adherence

One participant withdrew during the first week of tDCS sessions due to personal circumstances. Out of the planned 300 tDCS sessions, a total of 294 sessions were completed (Adherence = 98%). Six sessions were missed due to various reasons, including participants being occupied during the day or simply forgetting to conduct the session. The training session was well-received by all participants, and they demonstrated the ability to administer tDCS independently and safely after the training. Of the 31 participants, 30 completed the treatment protocol. Among them, 24 participants completed all sessions without missing any, while the remaining 6 participants missed one session each. The results are presented in Table 2.

### 3.2 Feasibility

Throughout the entire intervention period, we encountered technical issues a total of 23 times among all participants. There were 32 instances where participants faced challenges initiating the session due to impedance issues, yet no sessions were halted because of this. Additionally, no sessions were interrupted due to other technical issues or adverse effects. Out of the 29 participants, only 11 experienced any issues, and among these 11, only two required technical support via telephone, totaling three instances. Notably, no sessions were skipped due to adverse effects (Table 2).

### 3.3 Safety

In 284 sessions, only 4 participants experienced adverse effects. All reported side effects were mild and transient. Among them, one participant reported mild headaches during the first two sessions, another experienced dizziness during the initial sessions, and two participants described tingling or itching sensations under the electrodes during the first session. All reported side effects were mild, and participants did not express concern about them. In total, we identified mild side effects on 6 occasions, none of which required additional interventions (Table 2).

### 3.4 Perceived stress and anxiety

**Figure 3.**
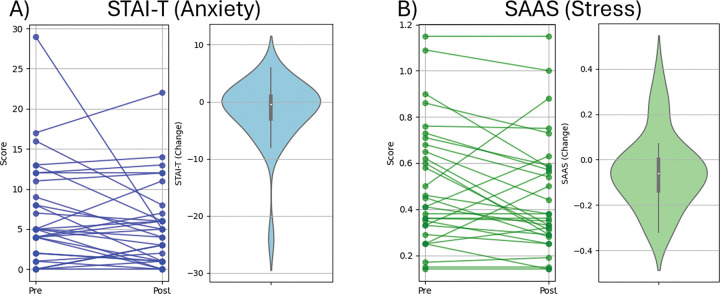
Changes in outcomes. A) Spaghetti plot showing individual STAI-T scores before and after the tDCS intervention (left), and violin plot illustrating the distribution of score changes (right). B) Spaghetti plot showing individual SAAS scores before and after the tDCS intervention (left), and violin plot illustrating the distribution of score changes (right).

Pre and post comparisons revealed a reduction in acute stress appraisal (z = 1.709, p = 0.045; see Fig. 3a), but not in the state sub-scale of the STAI-S (z = 0.672, p = 0.253, see Fig. 3b).

### 3.5 Psychophysiological outcomes

No significant differences were observed between baseline and post-treatment conditions in any of the physiological outcome measures. Specifically, both pupil amplitude and EDA measures—including raw amplitudes, model fit indices (R^2^), beta coefficients, and mean squared errors—showed no statistically significant changes following the intervention (all p-values > 0.05). However, EDA beta coefficients showed a tendency to be reduced in the post assessment, suggesting a diminished relation between circle distance and body physiological response. The amplitude of pupil diameter was significantly different (p < 0.01) between trials in which the circles eventually collided and triggered an electric shock, and those in which they approached but did not collide (see Supplementary Material).

## Discussion

The study aimed to investigate adherence, safety and feasibility of implementing a Home-Based tDCS protocol to modulate resilience to stress. As a second goal, we wanted to explore if this tDCS intervention could modulate response to stress events.

In line with Cappon (2022), our findings demonstrate excellent adherence to the home-based protocol, with 98% of scheduled sessions completed and only 2% skipped. Additionally, the dropout rate was minimal, with only 1 out of 31 participants discontinuing the study.

Remote supervision played a crucial role in ensuring and promoting adherence to the stimulation protocol, as highlighted by Riggs et al., (2018). However, most participants declined to continue daily monitoring because they felt confident following the offered training. Nonetheless, in these cases we only reduce the monitoring to a weekly phone call in order to mitigate the risk of misuse or overuse, aligning with safety recommendations (Castelo-Branco & Fregni, 2020). Importantly, our single training session proved to be concise yet effective, enabling participants to learn how to set up and self-administer tDCS sessions. Participants demonstrated a high level of proficiency in correctly setting up the device and administering the stimulation. Feedback from participants regarding the training session was generally positive. Many verbally expressed confidence in their ability to independently use tDCS, describing the procedure as easy to perform. This confidence was reflected in the minimal need for remote assistance, which occurred on only three occasions, involving just two participants. In line with Cappon (2022), there has been growing interest to employ home-based tDCS as an intervention for a wide array of clinical conditions. The methodology employed could be adapted to clinical contexts beyond intervention to modulate stress and anxiety. However, further studies are needed about the optimal training sessions and protocols for patients with diverse pathologies and employing different noninvasive brain stimulation devices and methods.

Our HB-tDCS intervention showed to be safe, as no serious adverse events and only rare, mild transient side effects were reported throughout the 284 home-based sessions. This finding aligns with previous studies investigating home-based tDCS interventions, such as the study conducted by Pilloni et al., (2022). To mitigate the risk of skin burns, our device, like other home-based tDCS devices mentioned by Bikson et al., (2020), has fixed stimulation parameter that cannot be changed by participants.

Following the HB-tDCS intervention, we observed a significant reduction in acute stress appraisal following stress induction. This might be due to the effects of tDCS on cognitive stress control and the autonomic system involving predominantly parasympathetic responses as suggested by Carnevali et al., (2020). On the other hand, no significant changes in anxiety levels were observed. Similarly, the effects of the stimulation were not evident in psychophysiological measures, possibly due to the short stimulation protocol, which is consistent with previous findings. For instance, Nishida et al. (2019) applied a single session of tDCS over the DLPFC in patients with major depressive disorder and reported no significant reduction in anxiety levels, as assessed by STAI (Nishida et al., 2019). These findings align with other studies employing different non-invasive brain stimulation (NIBS) techniques targeting the DLPFC for stress regulation, which have reported trends toward improved recovery responses to stressors, although these effects often fail to reach statistical significance (De Witte et al., 2020). It is difficult to reach definitive conclusions about the efficacy of tDCS for anxiety due to the heterogeneous nature of the existing literature (Stein et al., 2020). The lack of significant results in psychophysiological outcomes suggests that the intervention did not produce measurable changes in autonomic responses, as captured by pupil dilation or EDA-based markers.

In any case, our results on the efficacy of tDCS for stress and anxiety modulation must be interpreted cautiously. Our focus was of feasibility and safety. Importantly, the lack of a control group receiving sham stimulation prevents us from conclusively attributing the stress reduction to the tDCS intervention. It remains unclear whether the observed reduction is due to a “practice” effect from task repetition or a placebo effect. Other relevant considerations include the number of tDCS sessions administered. It is possible that an increased number of sessions would lead to observable changes in psychophysiological measures. Therefore, it would be of interest to replicate the current protocol with an extended stimulation schedule. Additionally, the moving-circles, stress-induction paradigm could be modified for greater sensitivity. In the present study, the number of instances when the circles touched and participants were delivered a mild electric stressor was only eight, while near-collision events—instances where the circles almost collided but ultimately did not—were also eight. Extending the duration or intensity of the paradigm could yield a larger dataset of stressor events, potentially enhancing statistical power and the significance of the observed effects.

In conclusion, HB-tDCS offers several advantages over traditional in-person protocols. By removing the need for frequent clinic visits, it reduces logistical barriers and enhances convenience for patients, particularly those residing in remote or underserved areas. Moreover, the personalized nature of HB- tDCS enables tailored intervention protocols, optimizing outcomes by accommodating individual variations in response to stimulation. While promising, the implementation of HB-tDCS necessitates careful consideration of ethical and safety implications. Ensuring suitable training and supervision for users, as well as establishing robust monitoring mechanisms, is paramount to mitigate risks and safeguard patient well-being.

Recent evidence indicates that inadequately supervised Home-Based tDCS (HB-tDCS) approaches may result in premature termination or a lack of necessary rigor, leading to suboptimal clinical outcomes and safety risks (Kumpf et al., 2023). However, this study demonstrates that home-based, remotely supervised tDCS interventions are feasible, safe, and potentially effective in modulating individuals’ stress regulation. Nevertheless, it is important to recognize that individuals with pathologies may face greater challenges in learning to use the device and completing the intervention. Further research is necessary to establish the efficacy and long-term outcomes of this type of intervention for stress reduction.

## Supplementary Material

Supplementary Files

This is a list of supplementary files associated with this preprint. Click to download.

• Suplementary.docx

## Figures and Tables

**Figure 1.**
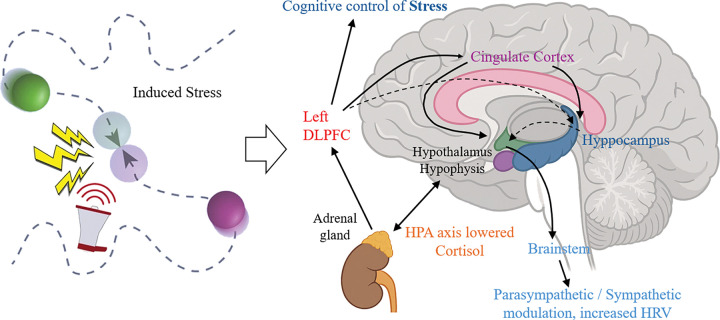
Description of the stress induction paradigm: The diagram, adapted from (Limbachia et al., 2021), illustrates the stress induction paradigm where two circles move randomly across the screen, and an mild electric shock is delivered to the participant when the circles collide. This figure also represents how the left DLPFC modulates the stress response, leading to lowered cortisol levels following the tDCS intervention, in line with Castelo-Branco & Fregni, (2020).

**Figure 2.**
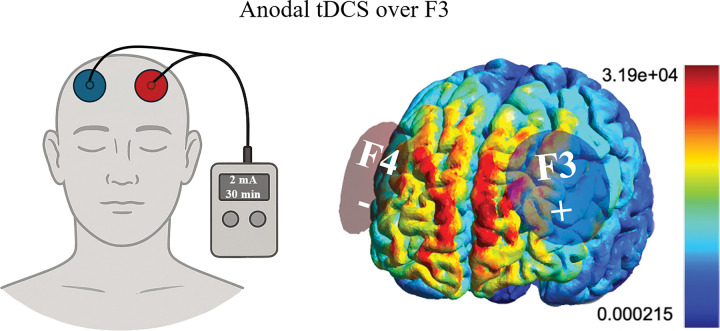
tDCS device setup with the anode positioned at F3 and the cathode at F4 (left). Simulation of the electric field magnitude (right) during stimulation of the prefrontal cortex with the anode at F3 and the cathode at F4 was simulated using SimNIBS (Saturnino et al., 2018).

**Table 1:** Demographic characteristics of participants.

Variable	Category	n	% / Mean (SD)
Age		–	57.68 (5.89)
Sex	Male	15.00	48.39
	Female	16	51.61
Education	Primary (≤ 8 years)	0	0.00
	Secondary (9–12 years)	6	19.35
	Superiors (≥ 13 years)	25	80.65

**Table 2:** Adherence, feasibility and types of side effects outcomes.

Adherence	Total	Percentage		
Complete sessions	294	98.0		
Skkiped sessions	6	2.1		
Treatment finished	30	96.8		
Treatment full completed	24	77.4		
Dropouts	1	3.2		
Feasibility	Total			
Issues during sessions	23			
Unallowed start due impedance	32			
Uncomplete sessions due impedance	0			
Skipped sessions due technical issues	0			
Skipped due adverse effects	0			
Participants who experimented issues	11			
Participants who needed technical support	2			
Techical support required	3			
Side effects	Mild	Moderate	Severe	Percentage of sessions
Headache	2	0	0	0.68
Neck Pain	0	0	0	0.00
Scalp pain	0	0	0	0.00
Sensations under electrodes	2	0	0	0.68
Sleepiness	0	0	0	0.00
Scalp burn	0	0	0	0.00
Scalp redness	0	0	0	0.00
Dizziness	2	0	0	0.68

**Table 3:** Repeated measures ANOVA results for each outcome measure related to pupil diameter and EDA.

Variable (DV)	Source	SS	DF	MS	F	p-unc	η^2^g	ε
Pupil Amplitude	PrePost	9157.73	1	9157.73	0.434	0.523	0.01	1
	Error	253250.35	12	21104.2				
EDA Amplitude	PrePost	0.227	1	0.227	0.41	0.529	0.011	1
	Error	11.05	20	0.553				
Pupil R^2^	PrePost	0.0038	1	0.0038	0.459	0.511	0.015	1
	Error	0.0982	12	0.0082				
EDA R^2^	PrePost	0.0001	1	0.0001	0.024	0.879	0	1
	Error	0.1237	20	0.0062				
Pupil MSE	PrePost	0.0003	1	0.0003	0.025	0.877	0.001	1
	Error	0.1341	12	0.0112				
EDA MSE (log)	PrePost	0.2655	1	0.2655	0.196	0.663	0.002	1
	Error	27.0752	20	1.3538				
Pupil Beta (EST)	PrePost	0.1188	1	0.1188	1.035	0.329	0.025	1
	Error	1.3767	12	0.1147				
EDA Beta (EST)	PrePost	0.0382	1	0.0382	3.224	0.088	0.023	1
	Error	0.2368	20	0.0118				
